# Primary Ciliary Dyskinesia: Phenotype Resulting From a Novel Variant of LRRC56 Gene

**DOI:** 10.7759/cureus.28472

**Published:** 2022-08-27

**Authors:** Badriah G Alasmari, Muhammad Saeed, Mohammed A Alomari, Mohammad Alsumaili, Ali M Tahir

**Affiliations:** 1 Department of Pediatrics, Armed Forces Hospital Southern Region, Khamis Mushait, SAU

**Keywords:** lrrc56, respiratory infection, primary ciliary dyskinesia, situs inversus totalis, a new variant, lrrc56 gene, paediatrics respiratory infection, primary ciliary dyskinesia (pcd)

## Abstract

Primary ciliary dyskinesia (PCD) involves cilia impairment, with resultant symptoms of repeated respiratory infections, sinusitis, and infertility. We report a seven-year-old boy of Arab ethnicity, with consanguineous parents, who was identified to have situs inversus totalis in neonatal life. There was a significant family history of ciliopathy as situs inversus totalis, infertility, and recurrent respiratory infections were noted in his two paternal uncles. From five months of age, the child started to have recurrent hospital visits due to respiratory infections. Infancy was marked by failure to thrive along with delay in achieving developmental milestones. Next-generation sequencing of known or potential ciliopathy genes revealed him homozygous for a novel mutation c.494T>C of the *LRRC56* gene, thus defining PCD as a potential cause of his features.

## Introduction

Primary ciliary dyskinesia (PCD) is a rare respiratory entity with an autosomal recessive inheritance. It characterizes motile cilia impairment, whether in structure or function. Motile cilia entail to upper and lower respiratory tract, fallopian tube, and sperm tail. Their pathology consequentially results in sinusitis, otitis media, repeated respiratory infections, as well as infertility [1]. Situs inversus is a usual phenomenon [2]. The reported prevalence of PCD is one in 2,200-40,000 live births [3]. The diagnostic dilemma is reflected by the absence of any "gold standard" diagnostic test for PCD [4]. Genetic studies can identify more than 70% of cases [5]. We report a case of a seven-year-old boy with PCD-defining features diagnosed with a novel homozygous mutation in the *LRRC56* gene.

## Case presentation

**Figure 1:**
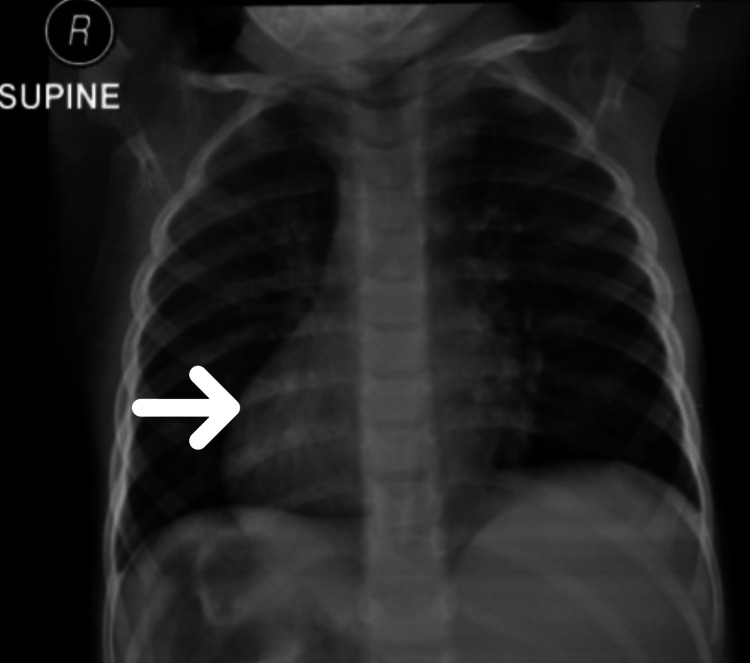
Chest X-ray (anteroposterior view) depicting dextrocardia (white arrow)

A seven-year-old boy with consanguineous parents of Arab ethnicity, delivered at term through cesarean section, had a history of five days of admission in the neonatal intensive care unit (NICU) due to transient tachypnea of the newborn. He was identified as a case of situs inversus totalis. He had an appreciable family history of ciliopathy (situs inversus totalis, infertility, and recurrent sinopulmonary infection) as his two paternal uncles were affected. Initially, he had an indolent course till five months of age when he had five days of inpatient care due to bronchopneumonia. Infancy was marked by failure to thrive, as he just weighed 5 kg on his first birthday. He was delayed in achieving developmental milestones. It was followed by recurrent admissions due to upper and lower respiratory tract infections. He had good cognitive functions with no dysmorphism. Apart from dextrocardia, systemic examination revealed no abnormality (Figure 1).

**Figure 2:**
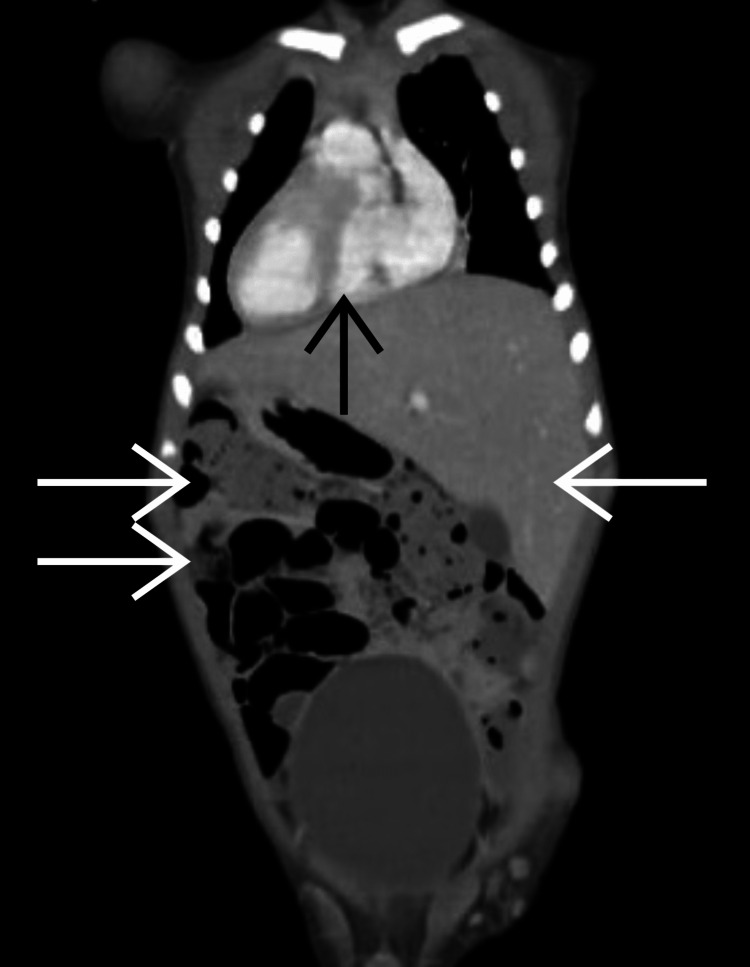
Coronal section of the abdomen and chest CT exhibiting situs inversus totalis (dextrocardia demonstrated by a black arrow and peritoneal organ inversion demonstrated by white arrows)

Blood counts and renal and liver functions all stayed within normal range. Barium study in infancy was consistent with moderate gastroesophageal reflux disease (GERD). CT scan exhibited situs inversus totalis with bilateral maxillary sinusitis (Figure 2).

Next-generation sequencing of known or potential ciliopathy genes revealed him homozygous for a novel mutation c.494T>C of the *LRRC56* gene. It was characterized by nucleotide exchange in exon 8 of the *LRRC56* gene, evolving as a missense variant c.494T>C, resultantly a non-conservative substitution of the evolutionary highly conserved amino acid leucine by protein at position 165 of the protein sequence (p.Leu165Pro).

The child was managed with supportive care and efficient chest physiotherapy, essentially during acute respiratory infections. Empirical antibiotics were given during acute pulmonary exacerbations. In addition to routine vaccination, an influenza shot was given annually. After the initial undesired course in terms of growth parameters, the child thrived well and exhibited catch-up growth after three years of age in lieu of extended nutritional support. The child is being followed regularly with no evidence of any bronchiectatic changes and demonstrating normal parameters of lung functions on spirometry.

## Discussion

PCD, a rare genetic disorder, remains the best-understood ciliopathy. It encompasses an established diagnostic pathway that has become well recognized. It confers a primary defect in the structure or functioning of cilia. PCD entails a dysfunctional mucociliary clearance with susceptibility toward chronic recurrent respiratory infections, male infertility, and laterality defects [6]. Being a genetically inherited disorder, ascertaining the genetic diagnosis of PCD early in the disease course can aid in extending appropriate care and management. Moreover, early diagnosis may result in the timely identification of potential complications. Addressing PCD with early diagnosis and holistic management reflects in better prognosis. Even though 50% of PCD cases have easily recognizable situs abnormalities and neonatal distress, only half get their diagnosis before the first birthday [7]. Situs inversus totalis with frequent respiratory infections should herald any clinician toward PCD. At least 36 genes have been linked to this heterogeneous genetic entity, which projects with autosomal recessive inheritance [8]. To the best of our knowledge, this missense alteration with c.494T>C has not been described in the literature or the database yet. The pathogenic character of the *LRRC56* missense variant c.494T>C (p.Leu165Pro) seems plausible in PCD etiology [9].

## Conclusions

In conclusion, we identified a novel pathogenic variant in the *LRRC56* gene, eventually linked to recurrent respiratory infection and significant growth restriction. This may expand the genetic canvas and drive us toward better personalized interventions in PCD. Better awareness may lead to diagnosis at an early stage, especially for genes that encode subtle ciliary phenotypic abnormalities, and will aid in establishing appropriate follow-up and introduction of careful management from early life. Further to this feat, improving the knowledge base of primary care physicians regarding PCD is essential and genetic counseling for patients and their families is equally desired.
